# Assessing zero-shot generalisation behaviour in graph-neural-network interatomic potentials

**DOI:** 10.1039/d5dd00103j

**Published:** 2025-09-30

**Authors:** Chiheb Ben Mahmoud, Zakariya El-Machachi, Krystian A. Gierczak, John L. A. Gardner, Volker L. Deringer

**Affiliations:** a Inorganic Chemistry Laboratory, Department of Chemistry, University of Oxford Oxford OX1 3QR UK chiheb.benmahmoud@chem.ox.ac.uk

## Abstract

With the rapidly growing availability of machine-learned interatomic potential (MLIP) models for chemistry, much current research focuses on the development of generally applicable and “foundational” MLIPs. An important question in this context is whether, and how well, such models can transfer from one application domain to another. Here, we assess this transferability for an MLIP model at the interface of materials and molecular chemistry. Specifically, we study GO-MACE-23, a model designed for the extended covalent network of graphene oxide, and quantify its zero-shot performance for small, isolated molecules outside its direct scope, as well as for examples of chemical reactions. Our work provides quantitative insight into the generalisation ability of graph-based MLIP models and, by exploring their limits, can help to inform future developments.

## Abstract 2

We explore to what extent a machine-learned interatomic potential trained for graphene oxide is applicable to isolated molecules and reactions in a ‘zero-shot’ setting.

## Graphical abstract

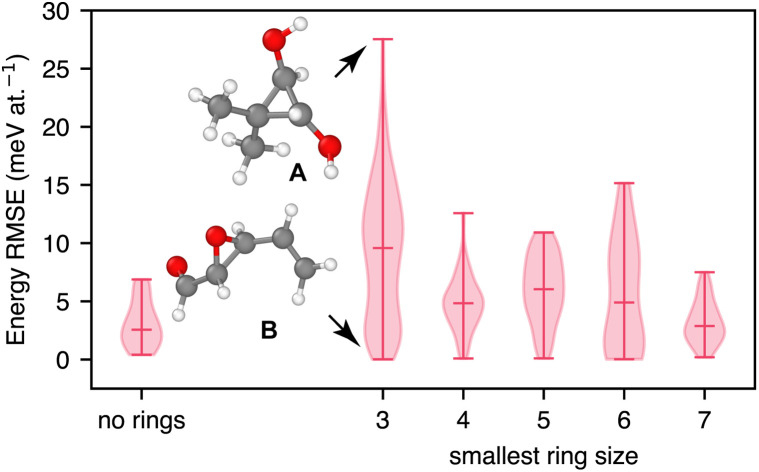

## Introduction

Machine-learned interatomic potentials (MLIPs) for atomistic simulations, trained on quantum-mechanical energy and force data, have advanced remarkably in recent years^1–3^ and now almost routinely allow researchers to address a wide range of questions in chemistry and materials science.^4–7^ Recently, MLIPs incorporating graph-based representations, commonly referred to as graph neural networks (GNNs),^8–12^ have emerged as cost-effective yet chemically rich models of atomic interactions. The favourable constant scaling of GNN-based MLIPs with the number of atomic species means that they are, in principle, able to cover many elements from across the Periodic Table all in a single model.^12–15^

The enhanced chemical versatility provided by GNNs has inspired the development of so-called “pre-trained”,^12^ “foundational”,^13^ or “universal”^15,16^ interatomic potentials. These models have been trained on large, structurally and chemically diverse datasets; they show promising baseline performance for a range of systems^17,18^ and thus provide a practical tool for starting computational projects, as well as a basis for fine-tuning.^19^ In the long run, one might want to employ these pre-trained MLIPs “as is”, in a zero-shot manner, without additional training or adaptation. Zero-shot performance also yields an important indication of how well the underlying model generalises to unseen tasks and chemistries. Understanding and improving the zero-shot behaviour of MLIPs is therefore an important challenge.

Herein, we study the zero-shot generalisation behaviour of GO-MACE-23 (ref. 20), an MLIP model that was initially developed specifically for graphene oxide (GO). Conceptually, GO bridges the gap between pristine graphene and organic chemistry: its structural landscape involves a variety of bonding motifs from sp^2^ carbon sheets to oxygen-rich domains and reactive edge sites.^21^ We test whether this structural and chemical complexity may serve as a basis for transferability (albeit initially we thought of GO-MACE-23 as a single-purpose MLIP!), subjecting GO-MACE-23 to a range of out-of-domain benchmarks, from energetics to high-temperature molecular-dynamics (MD) simulations of chemical reactions. In this way, our present study explores: (i) the role of a chemically rich training dataset in building robust and generalisable MLIPs;^22^ (ii) the importance of GNN-based architectures in doing so; and (iii) the question whether GO-MACE-23 could form a starting point for foundational MLIPs bridging materials and molecular chemistry. Data and code supporting this work are publicly available (see “Data availability” statement below).

## Methodology

### The GO-MACE-23 and MACE-OFF24 models

We focus on the GO-MACE-23 model, which was built using the MACE architecture^10,11^ together with a bespoke data-generation protocol.^20^ Initial training data were generated “from scratch” using CASTEP + ML^23^ (accelerating *ab initio* MD through on-the-fly fitting of GAP models^24^), and then largely augmented through subsequent iterative training from MD trajectories driven by intermediate versions of MACE models. Over multiple iterations, configurations with functionalised edges, involving hydroxyl (–OH), aldehyde (–CHO), and carboxylic acid (–CO_2_H) moieties, were added to ensure good coverage of the structural and chemical features that might be expected to appear in a “real-world” GO sheet. Training labels, *viz.* total energies and forces, were obtained from density-functional-theory (DFT) computations performed with the plane-wave software CASTEP^25^ using on-the-fly generated pseudopotentials, the Perdew–Burke–Ernzerhof (PBE) exchange–correlation functional,^26^ and a plane-wave energy cutoff of 550 eV. An overview of the GO dataset is available in the SI.

As a baseline for current practice in modelling organic molecules, we choose two variants of the MACE-OFF family of MLIPs:^27^ the “large” version of MACE-OFF23 commonly referred to as MACE-OFF23(L), which is trained on the SPICE dataset of molecular data version 1,^28^ and the “medium” version of MACE-OFF24 commonly referred to as MACE-OFF24(M), which is trained on the SPICE dataset version 2.^29^ MACE-OFF24(M) is more similar to GO-MACE-23 in terms of architecture, with the exception of the radial cut-off: 3.7 Å for GO-MACE-23 and 6.0 Å for MACE-OFF24. More details about the hyperparameters of the GNNs used in this work are provided in the SI. In the remainder of this work, we refer to MACE-OFF23(L) simply as MACE-OFF23 and to MACE-OFF24(M) as MACE-OFF24. In using MACE-OFF24 models as benchmarks, it is important to note the different DFT levels of theory compared to GO-MACE-23 the SPICE labels were obtained using DFT with the ωB97M-D3(BJ) exchange–correlation functional^30,31^ and the def2-TZVPPD basis set.^32,33^

### Benchmark data

We carry out numerical experiments using the revised version of the MD17 dataset (rMD17)^34^ as well as the QM7-X dataset.^35^ We select the 6 molecules from rMD17 that only contain the elements C, H, and O—the only ones in the GO dataset, and thus the only ones that GO-MACE-23 and other models directly fitted to its dataset can handle. For each molecule, we randomly select 1000 configurations from the available trajectories. The rMD17 labels were obtained in the original work using the PBE functional and the def2-SVP basis set.^26,32^ As for QM7-X, we randomly choose 100 configurations from each of the 6 most common chemical formulae that only include C, H, and O.

The other test sets used in the present study are generated either by running MD simulations in the *NVT* ensemble or by relaxing molecules. In both cases, we use GO-MACE-23 to perform these tasks. We compute reference data using DFT, matching the settings for GO-MACE-23 and MACE-OFF24, where applicable. For comparison to GO-MACE-23 labels are obtained from CASTEP by placing the molecules in large periodic cells (>20 Å). For MACE-OFF24 compatible labels are obtained using the Atomic Simulation Environment (ASE)^36^ Python interface of Psi4,^37^ version 1.4.

### Data overlap between molecules and graphene oxide

**Fig. 1:**
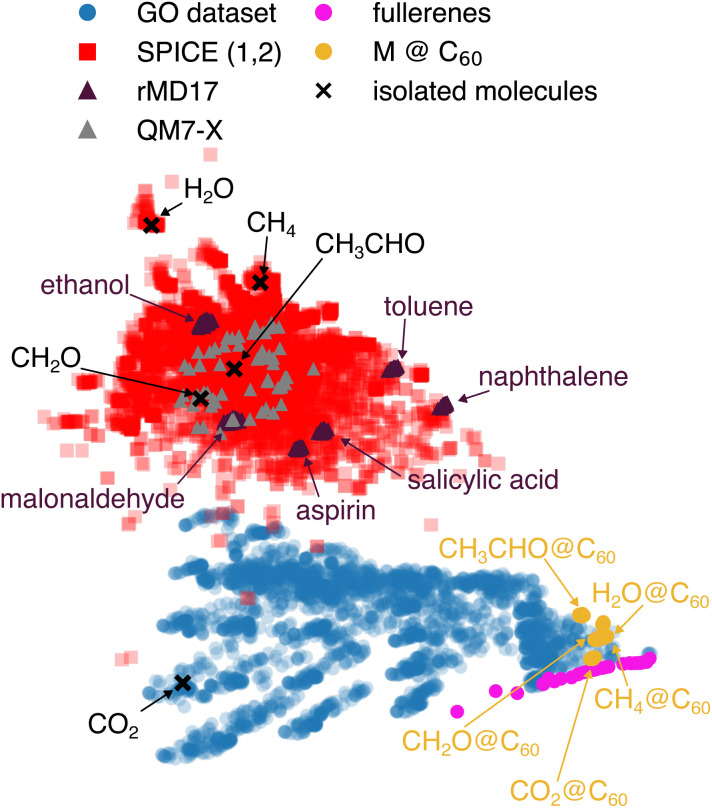
Visualising the structural and chemical space explored in the present study. We show a two-dimensional embedding of the MACE descriptor trained on the GO dataset,^20^ using principal component analysis. The points of the map correspond to the training set of GO-MACE-23 (blue), molecules containing C, H, and O atoms, representing ≈5% of the SPICE (version 1 and 2) datasets^28^ (red), selected configurations from rMD17 trajectories^34^ (purple) and the QM7-X dataset^35^ (grey), a series of fullerenes with sizes ranging between 20 and 100 (magenta), five molecules encapsulated in C_60_ fullerene cages (yellow), and the same molecules in vacuum (black crosses).

Before benchmarking GO-MACE-23 it is important to set performance expectations based on the similarity of the various test sets and the GO training set. In Fig. 1, we present a two-dimensional embedding, from principal component analysis (PCA), of the average atomistic features per snapshot as learned by GO-MACE-23. The use of average features eliminates the system-size dependence of the descriptors. In the map of Fig. 1, static rMD17 molecules lie outside the scope of the training data (blue), but fall within the SPICE dataset domain (red), which constitutes the training data of MACE-OFF24. We should thus expect MACE-OFF24 to outperform GO-MACE-23 for static molecules. Fullerenes (magenta) and encapsulated molecular species (“M @ C_60_”, yellow) are located on the outskirts of the GO region of the map—this is unexpected at first glance, as fullerenes are not part of the GO training data. However, some of their key characteristics can be learned from the GO backbone.

### Zero-shot performance of GO-MACE-23

In this section, we evaluate the performance of GO-MACE-23 in predicting the energies and forces of small molecules, as well as vibrational spectra. Throughout this section, we use the terms “error” and “root mean square error” (RMSE) interchangeably.

### Numerical performance for rMD17 and QM7-X

A common starting point in evaluating MLIP performance is to test prediction errors for energies and forces. These tests can be more complex than they look at first glance, because their outcome will strongly depend on the type of data used for testing (see, *e.g.*, ref. 38–44). In the present work, we are interested in zero-shot generalisability (without further modification of the model), which we here test by changing the application domain from extended GO structures to isolated small molecules.

**Fig. 2:**
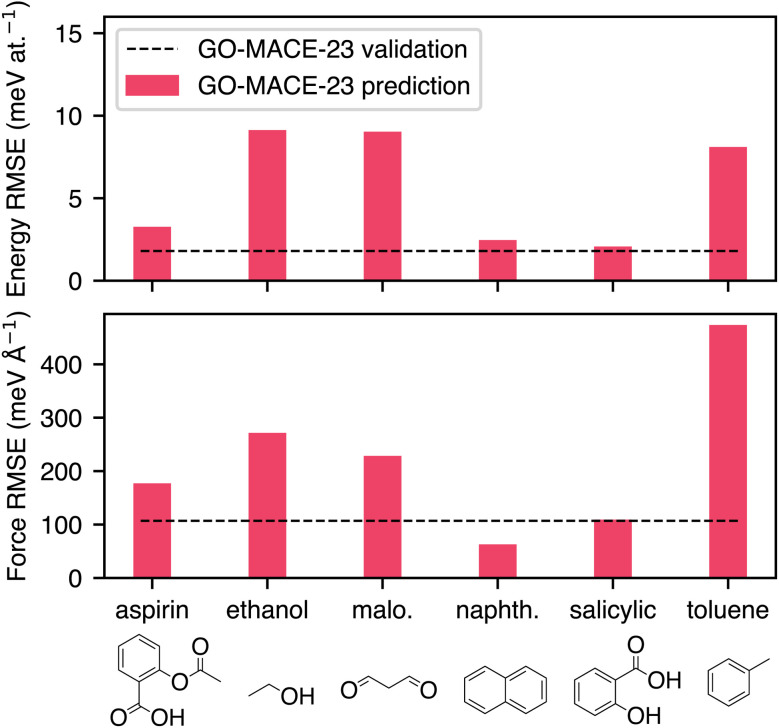
Energy and force errors on six trajectories from the revised MD17 dataset for GO-MACE-23. The bars represent the RMSE of quantities between GO-MACE-23 predictions and its DFT level of theory. The dashed line is the internal validation error of GO-MACE-23.

We begin our series of zero-shot tests by evaluating the performance of GO-MACE-23 for the relevant trajectories from the rMD17 dataset. In Fig. 2, we summarise prediction errors on total energies and atomic forces relative to the recomputed QM targets using the same level of theory as that of GO-MACE-23. We obtain energy RMSE values below the often-quoted “chemical accuracy” of 1 kcal mol^−1^ or ≈40 meV at.^−1^. However, these errors can be significantly higher than the model's internal validation error for GO (1.8 meV atom^−1^ for energies and 109 meV Å^−1^ for forces, shown as dashed lines in Fig. 2). For aspirin, naphthalene, and salicylic acid from the rMD17 dataset, prediction errors of GO-MACE-23 for both energies and forces are compatible with the MLIP's validation errors on the GO dataset.

**Fig. 3:**
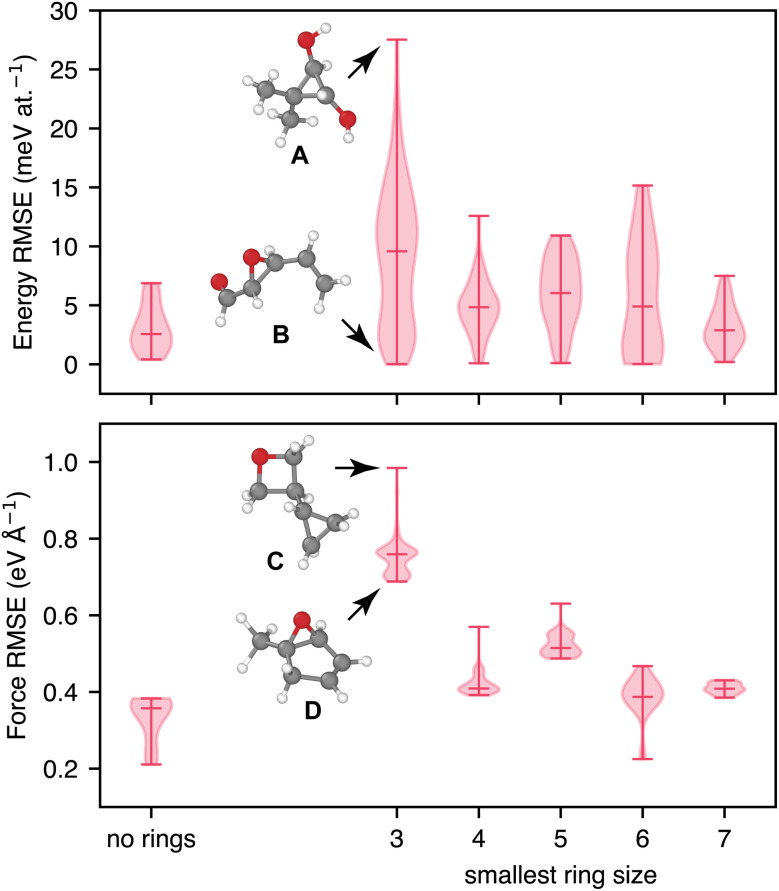
As Fig. 2, but for configurations from the QM7-X dataset. We group the results based on the smallest ring found in the respective molecule. Four examples are highlighted: A and B, showing the highest and lowest energy error among all structures where the smallest ring is 3-membered, and C and D, showing the highest and lowest force error among those.

We next study the performance of GO-MACE-23 for molecules drawn from the more diverse QM7-X dataset (Fig. 3). Grouping the energy and force prediction errors according to the smallest ring size in any molecule (or the absence of any rings) reveals that the model's performance appears to correlate to some extent with the size and chemical nature of the smallest ring in the system. We select molecules containing 3-membered rings to illustrate this point: structure A has a cyclopropyl (C_3_) ring, unlikely to be present in a well-annealed GO structure, and shows the highest energy RMSE of all selected structures containing any three-membered ring; by contrast, the 3-membered ring in B is an epoxy (C–O–C) moiety, a well-known structural motif in GO,^20,21^ and this molecule has the lowest energy RMSE among those characterised in Fig. 3. Similar arguments can be made for the molecules with the highest and lowest force error, respectively: C contains a 3-membered cyclopropyl as well as a 4-membered oxetane ring, whereas D again shows an epoxy group as the 3-membered structural unit.

We note that despite these relatively large numerical errors, GO-MACE-23 is still robust: it yields stable MD trajectories of all molecules from rMD17 and QM7-X in the *NVT* ensemble at *T* = 500 K for 1 ns.

This evaluation highlights the importance of contextualising zero-shot performance of pretrained ML models across datasets. Most of the force prediction errors stem from the presence of under-represented geometries in the training set, as suggested by Fig. 2 where molecules with structural motifs resembling those in a GO sheet are better captured by GO-MACE-23 reinforcing the importance of dataset choice for generalisability. In the following subsection, we analyse one of these cases in detail: toluene from rMD17. It is worth noting that, although we were able to recompute DFT labels for all test molecules in our work, this is not a typical scenario. In many cases, comparisons are made across different levels of theory, meaning that systematic errors arising from the labels are entangled with, but distinct from, the model's own uncertainties. This underscores the importance of robust contextual analysis in ML model evaluation.

### Toluene as a special case

**Fig. 4:**
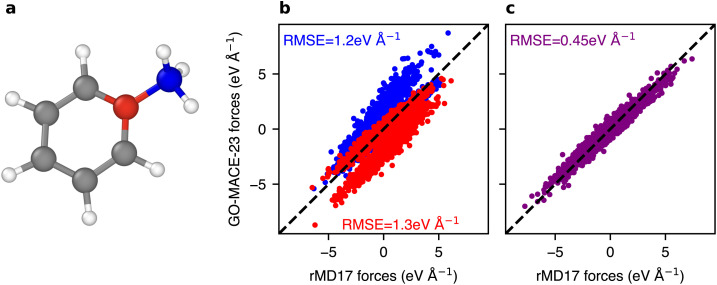
(a) Visualisation of a toluene molecule obtained using OVITO.^45^ Red- and blue-coloured atoms are carbon atoms part of the aromatic ring and the attached methyl group, respectively. (b) Force components parity plot of the DFT-computed and GO-MACE-23-predicted forces for the carbon atoms labelled red and blue in panel (a). (c) Force parity plot of the sum of forces of the red- and blue-labelled carbon atoms.

To better understand the performance limits of GO-MACE-23 we analyse the errors for toluene in more detail, as it exhibits the highest force prediction RMSE among all 6 rMD17 molecules considered here. Fig. 4 summarises our approach to exploring possible sources of error. The toluene molecule contains an aromatic carbon atom directly bonded to an sp^3^ carbon atom in a methyl group (–CH_3_), coloured in red and blue in Fig. 4a, respectively. These two carbon atoms have the highest overall force errors exceeding 1.2 eV Å^−1^ (Fig. 4b). The high force errors on these specific atoms indicate that GO-MACE-23 cannot faithfully model their behaviour, due to the under-representation of similar atomic environments in the training set.

Most current MLIPs (including the MACE architecture) describe the total energy of a chemical system as a sum of atomic energies, following ref. 46 and 24. While this decomposition is useful for training and extrapolating ML models, it is not inherently physical and has no direct counterpart in a quantum-mechanical computation: so it is possible for the MLIP to reproduce the global behaviour without capturing the expected local energy distribution. This issue is evident in the present case of toluene (Fig. 4c): the combined error for the sum of the forces is only one-third of the individual force-component errors. The predicted atomic energies confirm this limitation (Fig. S2): the “red” atom of the aromatic ring has the lowest predicted atomic energy of all the carbon atoms, while the “blue” atom of the methyl group has the highest. When averaging the energies of these two atoms, the methyl carbon and its direct neighbour have the lowest local energy across the randomly selected 200 snapshots in the trajectory (Fig. S2). The atomic decomposition ansatz provides a partial explanation in this case. More generally, further work is necessary to fully understand the local predictions of MLIPs, and steps towards this goal have been made.^47–49^

### Vibrational spectra

**Fig. 5:**
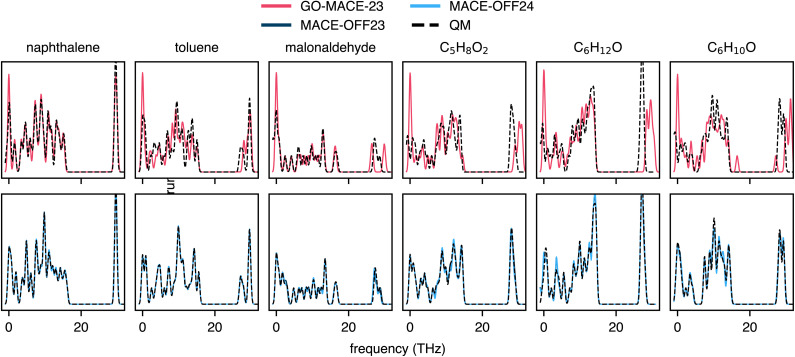
Molecular vibrational spectra computed with MLIPs (solid lines) and DFT (“QM”, dashed lines) for GO-MACE-23-relaxed naphthalene, toluene, malonaldehyde, C_5_H_8_O_2_, C_6_H_12_O, and C_6_H_10_O molecules. The upper row characterises the out-of-domain performance of GO-MACE-23 (red). The lower row shows the performance of MLIPs trained for molecules, *viz.* MACE-OFF^27^ (dark and light blue, visually indistinguishable). Note that the DFT data have been computed at the level corresponding to the training data of the respective MLIP model; the DFT data in the upper and lower rows are therefore slightly different.

The vibrational spectrum—which provides information about bending, twisting, stretching, *etc.*, of individual bonds—is a fingerprint of a molecule (and experimentally accessible), and reproducing it accurately is therefore an important test for an MLIP. To assess the ability of GO-MACE-23 to predict vibrational spectra, we focus on three molecules from the rMD17 dataset: naphthalene and toluene representing the best and worst force predictions, respectively (*cf.*Fig. 2), and malonaldehyde as an example of a molecule without a 6-membered aromatic ring (the principal structural fragment of graphene). We also include one conformer each representing C_5_H_8_O_2_, C_6_H_12_O, and C_6_H_10_O from the QM7-X dataset. We start by selecting a random snapshot from the six subsets, then relax the molecules using GO-MACE-23. The force errors for the relaxed structures are 0.05, 0.32, and 0.22 eV Å^−1^ for naphthalene, toluene, and malonaldehyde, respectively, and 0.31, 0.66, and 0.33 eV Å^−1^ for the selected C_5_H_8_O_2_, C_6_H_12_O, and C_6_H_10_O structures, respectively. Then, we compute the vibrational spectra with the MLIP and DFT at the corresponding level, using finite displacements, with phonopy.^50,51^ We present the resulting spectra in the upper panels of Fig. 5. The GO-MACE-23-predicted spectra agree qualitatively with their DFT counterparts, and the quality of the prediction correlates well with the model's force accuracy. The low-frequency modes, in particular, are well reproduced, while the accuracy decreases for the high-frequency modes. Additionally, we relaxed these molecules using DFT and report, in the SI, the root-mean-square displacement between geometries optimised with DFT and GO-MACE-23. We note that the discrepancies are relatively high, ranging between 0.17 Å and 0.28 Å. A recent study in ref. 52 suggests that these discrepancies may arise from a softened potential-energy surface near the relevant snapshots, which could explain the reduced accuracy for high-frequency modes.

We compare GO-MACE-23 to MACE-OFF23 and MACE-OFF24, two molecular MLIP models trained on different versions of the SPICE molecular dataset (see Methodology section). We compute the vibrational spectra on the GO-MACE-23-relaxed molecules using MACE-OFF24 and their corresponding DFT level of theory. The force errors of MACE-OFF23 are 0.003, 0.002, 0.016, 0.023, 0.015, and 0.008 eV Å^−1^ for naphthalene, toluene, malonaldehyde, C_5_H_8_O_2_, C_6_H_12_O, and C_6_H_10_O respectively. The force errors of MACE-OFF24 are 0.005, 0.003, and 0.005, 0.033, 0.03, 0.016 eV Å^−1^ for naphthalene, toluene, malonaldehyde, C_5_H_8_O_2_, C_6_H_12_O, and C_6_H_10_O respectively. We report the spectra in the lower panels of Fig. 5. As shown in Fig. 1, the rMD17 molecules are structurally similar to the training domain of the MACE-OFF24 models, which explains the models' high accuracy in predicting atomic forces. As a result, both MACE-OFF24 models produce more accurate vibrational spectra, reproducing both high- and low-frequency modes.

### Fullerenes and encapsulated molecules

We use a series of fullerene molecules as another benchmark to quantify the transferability of GO-MACE-23 (and MACE-OFF24). The smallest fullerene is C_20_, containing only 5-membered rings of carbon atoms and no 6-membered ones; consequently, its curvature is large. Yet, the fullerene was found to be the most stable C_20_ isomer using MP2 computations.^54^ Larger fullerenes are structurally closer to graphene and graphite, and should therefore be closer to the training domain of GO-MACE-23 (*cf.*Fig. 1).

**Fig. 6:**
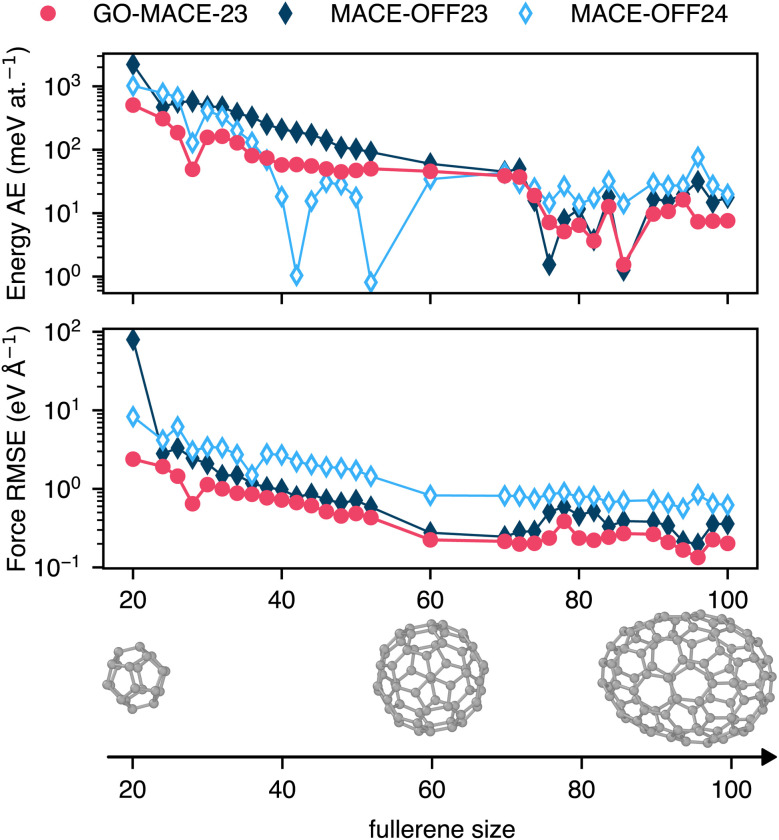
Evolution of the prediction errors of the per-atom energy and forces of fullerenes, obtained from ref. 53, of sizes between 20 and 100 atoms computed with GO-MACE-23 and its corresponding DFT level of theory (red), and MACE-OFF and their corresponding DFT level of theory (dark and light blue). Similar to Fig. 5, lines represent the ML predictions, and the dashed lines represent the QM reference calculations. The rendered images show three fullerenes: C_20_, C_60_, and C_100_.

We first test the stability of GO-MACE-23 in generating MD trajectories for fullerenes. We run *NVT* simulations for C_20_, C_60_, and C_100_ for 1 ns, targeting *T* = 500 K. We find that GO-MACE-23 maintained structural integrity throughout the simulations, producing stable trajectories without signs of unphysical distortions. Both GO-MACE-23 and the MACE-OFF24 variants reproduce the general trend of growing stabilisation with fullerene size (Fig. 6). Prediction errors are highest for the smaller fullerenes, with energy errors of >100 meV at.^−1^, and force RMSE >2 eV Å^−1^, likely due to their high curvature. For C_60_, the energy errors decrease to around 50 meV at.^−1^ for all MLIPs, and force errors to around 250 meV Å^−1^ for GO-MACE-23 and MACE-OFF24. For small fullerenes (<60 carbon atoms), GO-MACE-23 performs better than both MACE-OFF24 models: we presume that this is due to the fact that it has encountered some curved graphene sheets (SI), including various odd-membered rings, during training. Note, however, that the latter are only a small fraction of the training data: the ring-size distribution in the GO-MACE-23 dataset is 1 : 600 for 5 : 6-membered rings. MACE-OFF24 notably outperforms both GO-MACE-23 and MACE-OFF23 for fullerenes with 42 and 50 atoms. Analysis of the overlap between atomic environments in the SPICE datasets and the fullerene set (Fig. S4) shows that this overlap is limited to smaller fullerenes (<40 atoms for SPICE 1 and <50 atoms for SPICE 2), suggesting that the strong performance of MACE-OFF24 cannot be explained solely by training–test similarity. As a further test, we use GO-MACE-23 and both MACE-OFF24 models to calculate the vibrational spectra of two fullerenes, C_20_ and C_60_, using the same protocol as for the rM17 and QM7-X molecules (Fig. S5). We find that GO-MACE-23 yields good accuracy compared to its DFT reference, while both MACE-OFF24 models fail to reproduce the spectrum of C_20_.

**Fig. 7:**
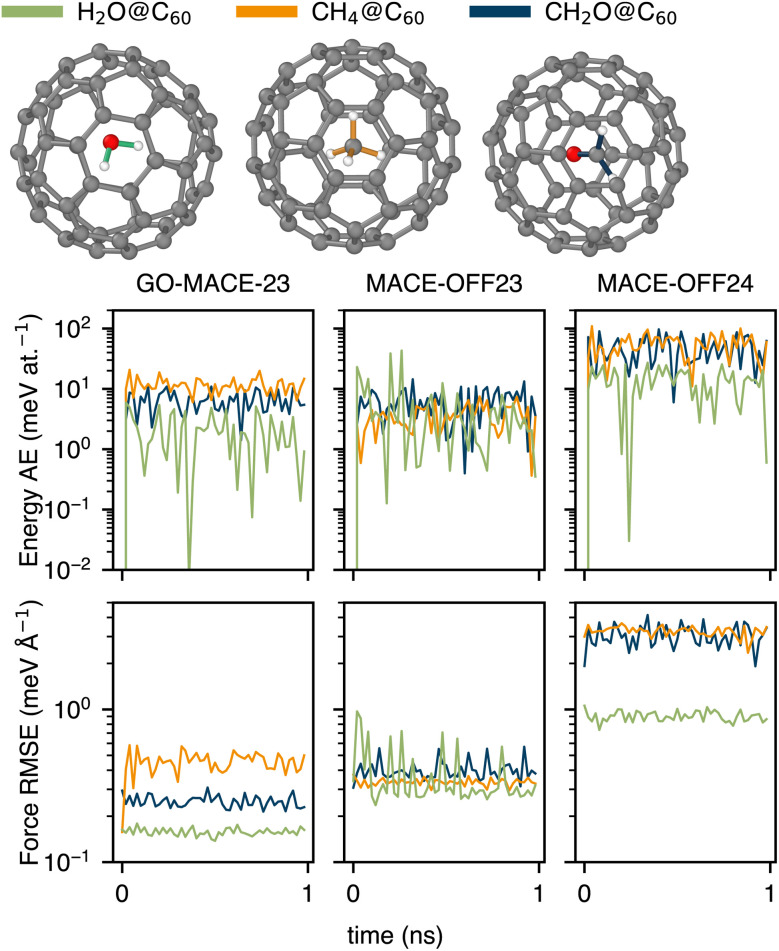
Evolution of energy and force RMSE between GO-MACE-23 predictions and the corresponding DFT level of theory (left column), as well as between both MACE-OFF variants and their respective DFT levels of theory (middle and right columns). The errors are calculated from 1 ns trajectories at 500 K for H_2_O, CH_4_, and CH_2_O enclosed in a C_60_ fullerene. The trajectories are driven by GO-MACE-23.

In a recent study, Vyas *et al.* showed how formaldehyde (CH_2_O) can be inserted into a C_60_ molecule by subsequent organic reaction steps,^55^ expanding on existing work on endohedral fullerenes.^56,57^ In the context of the present work, we show in Fig. 7 three case studies that have been discussed in the literature: encapsulated water (written as “H_2_O@C_60_”),^58^ encapsulated methane (“CH_4_@C_60_”),^59^ and encapsulated formaldehyde (“CH_2_O@C_60_”).^55^

We use GO-MACE-23 to drive long MD trajectories of the three species in the *NVT* ensemble at *T* = 500 K, for 1 ns with a 0.5 fs timestep. Such simulations can be challenging test cases,^60^ especially given the fusion temperature of C_60_ is estimated to be around 550 K.^61^ We re-label snapshots from these MD trajectories with GO-MACE-23 and its corresponding DFT method, as well as MACE-OFF24 and its corresponding DFT method. In Fig. 7, we show the errors, expressed as absolute error (AE) values for energies and RMSE for forces. Both MLIPs exhibit similar energy prediction errors, with GO-MACE-23 performing better for the larger encapsulated molecules, and MACE-OFF23 for H_2_O@C_60_. However, GO-MACE-23 consistently yields lower force prediction errors across all of the test cases. This poorer performance of MACE-OFF23 and MACE-OFF24 may be attributed to the fact that fullerenes and encapsulated molecules are not present within the two versions of the SPICE training set. Additionally, GO-MACE-23 has encountered small molecules, such as CO and H_2_O, near GO surfaces in its training data. Also, it is possible that GO-MACE-23 is accessing regions of configurational space that would be deemed unphysical by MACE-OFF24. To test this hypothesis, we run the same MD trajectories with MACE-OFF23 instead of GO-MACE-23 (SI). Of those simulations, only that for CH_2_O@C_60_ failed after the first timestep. We find that GO-MACE-23 more accurately reproduces the energies and forces for H_2_O@C_60_, whereas MACE-OFF23 performs better for CH_4_@C_60_. These results partially support the hypothesis that each MLIP explores regions of configurational space that are less well covered by the other MLIPs.

In the SI, we show two additional cases of encapsulated molecules, *viz.* CO_2_ and acetaldehyde, the heavier homologue of CH_2_O. Acetaldehyde is a challenging test case for GO-MACE-23, and has most likely not been seen during training (*cf.*Fig. 1). It is a thought experiment, of course, for the time being.

## Experiments

Beyond the zero-shot performance evaluation so far, we carry out additional numerical experiments. These explore aspects of MLIP fitting methodology and provide an initial test for descriptions of gas-phase fragmentation reactions.

### Model choice (I): effect of equivariant messages

The MACE architecture underlying GO-MACE-23 incorporates both invariant hidden features and equivariant hidden features of rank *L* = 1. In MACE, max *L* denotes the maximum degree of spherical harmonics used in the equivariant message-passing layers. It controls the complexity of the geometric information that the model can learn. For example, max *L* = 0 refers to an invariant model that can only capture isotropic features, and values of max *L* = 0 refer to an equivariant model encoding vectorial (and tensorial) information. To test the role of the equivariant features, we trained two modified versions of the model by varying MACE's internal symmetry rank. Specifically, we trained an invariant model by setting the highest rank of the internal features to max *L* = 0, and a higher-order equivariant model by setting max *L* = 2. This allows us to explore the possible correlation between the physical symmetries of an MLIP and its out-of-domain performance. Despite the fact that equivariant components can be included in MACE, the forces are computed by automatic differentiation of the total energy. All MACE models are trained with the same protocol as GO-MACE-23, as detailed in the SI.

In Table 1, we compare the performance of MACE models with different maximum rank, *viz.* max *L*∈{0, 1, 2}. We train each model on 4 splits of the GO dataset, and compute prediction errors and uncertainty estimates, as standard deviation over the 4 splits, for all relevant rMD17 and QM7-X molecules. We notice that the original GO-MACE-23 model (max *L* = 1) does not systematically outperform its invariant counterpart (max *L* = 0). For example, the invariant model yields better energy predictions for toluene, aspirin, malonaldehyde, C_5_H_10_O_2_, and C_6_H_8_O, as well as better force predictions for C_5_H_6_O_2_, compared to GO-MACE-23. A similar trend is observed when comparing energies predicted by GO-MACE-23 and the max *L* = 2 MACE model. The force prediction errors are comparable within their uncertainties. Regardless of the benchmark reference calculation, we observe no clear correlation between max *L* and model performance, suggesting that equivariance and symmetry preservation play a limited role in generalisation for these domains. Particularly notable cases are toluene, C_6_H_10_O, and C_6_H_8_O, where GO-MACE-23 is the worst-performing model of the three, in terms of total-energy prediction.

**Table 1:** Energy and force prediction RMSE as a function of the maximum rank of the equivariant hidden messages in the MACE architecture for trajectories from the rMD17 dataset and randomly selected structures from the QM7-X dataset (100 for each of the 6 given chemical formulae). “Malo.” stands for malonaldehyde and “Naphth.” for naphthalene. The lowest RMSE values for each molecules are highlighted in bold

max *L*	Energy RMSE (meV at.^−1^)			Force RMSE (eV Å^−1^)		
	0	1	2	0	1	2
Aspirin	4.6 ± 0.4	4.7 ± 1.0	**3.2 ± 0.6**	0.27 ± 0.01	0.21 ± 0.02	**0.19 ± 0.01**
Ethanol	9.3 ± 0.9	**8.7 ± 1.3**	8.8 ± 0.5	0.33 ± 0.03	0.32 ± 0.03	**0.27 ± 0.02**
Malo.	9.6 ± 2.1	10.2 ± 3.0	**8.4 ± 0.7**	0.21 ± 0.03	0.20 ± 0.01	**0.17 ± 0.01**
Naphth.	2.4 ± 1.0	**1.3 ± 0.5**	**1.3 ± 0.3**	0.09 ± 0.01	**0.06 ± 0.01**	0.07 ± 0.01
Salicylic	2.5 ± 0.6	**2.1 ± 0.4**	2.8 ± 0.9	0.10 ± 0.01	0.10 ± 0.01	**0.09 ± 0.00**
Toluene	5.9 ± 3.3	8.3 ± 3.8	**4.3 ± 1.7**	0.22 ± 0.05	0.17 ± 0.01	**0.15 ± 0.02**
C_6_H_10_O	25.5 ± 4.8	29.0 ± 3.4	**24.3 ± 2.4**	0.63 ± 0.05	0.62 ± 0.05	**0.50 ± 0.06**
C_5_H_8_O_2_	34.3 ± 7.6	34.5 ± 2.9	**34.0 ± 5.2**	0.46 ± 0.02	0.45 ± 0.04	**0.38 ± 0.04**
C_6_H_8_O	**44.8 ± 7.5**	56.5 ± 14.9	46.3 ± 6.7	0.56 ± 0.05	0.45 ± 0.05	**0.43 ± 0.06**
C_6_H_12_O	29.8 ± 12.0	33.7 ± 18.1	**15.7 ± 3.8**	0.49 ± 0.04	0.43 ± 0.06	**0.33 ± 0.04**
C_5_H_10_O_2_	**22.0 ± 2.5**	24.2 ± 3.9	32.4 ± 6.2	0.43 ± 0.02	0.41 ± 0.03	**0.36 ± 0.02**
C_5_H_6_O_2_	51.6 ± 6.2	**35.5 ± 10.0**	40.1 ± 7.0	0.43 ± 0.01	0.45 ± 0.02	**0.41 ± 0.05**

### Model choice (II): other GNN architectures

To further investigate the effect of design choices made for several popular GNNs on their generalisability, we trained multiple models on the GO-MACE-23 training dataset, using the universal interface graph-pes.^62^ Particularly, we used the PaiNN,^63^ TensorNet,^64^ and NequIP^9^ architectures. Details about hyperparameters, training protocol, and validation errors on the GO-MACE-23 dataset are provided in the SI.

Table 2 shows that GO-MACE-23 as well as re-fitted TensorNet and NequIP models generally yield low RMSE on most molecules considered. For instance, among the architectures in Table 2, NequIP achieves low energy errors on aspirin and malonaldehyde, whereas TensorNet performs best for toluene. Meanwhile, GO-MACE-23 has the lowest errors in force predictions for ethanol and naphthalene. These variations show that even closely related equivariant models can extract distinct mappings from the same data, influenced by subtle differences in model design and hyperparameters. We also compute the vibrational spectra of rMD17 and QM7-X molecules (*cf.* SI). We find that all of these GNNs reproduce the low-frequency spectrum with good accuracy, but the accuracy decreases substantially in the high-frequency regime.

Energy and force prediction RMSE different GNN architectures trained on the GO dataset, evaluated for structures from the revised MD17 dataset and QM7-X, as in Table 1. Errors are computed with respect to the DFT level of theory of the GO dataset. Malo.” stands for malonaldehyde and ”Naphth.” for naphthalene. The lowest RMSE values for each molecules are highlighted in bold

**Table:** 

	Energy RMSE (meV at.^−1^)			
	GO-MACE-23	TensorNet	NequIP	PaiNN
Aspirin	4.7 ± 0.6	9.2 ± 1.2	**4.1 ± 0.3**	8.2 ± 1.0
Ethanol	**8.7 ± 0.7**	23.9 ± 4.1	12.3 ± 0.5	16.9 ± 1.9
Malo.	10.2 ± 1.7	21.0 ± 5.3	**8.7 ± 0.8**	19.9 ± 2.6
Naphth.	**1.3 ± 0.3**	4.9 ± 1.1	3.4 ± 0.9	10.1 ± 1.8
Salicylic	**2.1 ± 0.2**	10.4 ± 2.4	3.1 ± 0.6	19.4 ± 2.5
Toluene	8.3 ± 2.2	15.1 ± 1.5	**8.1 ± 1.8**	30.5 ± 6.4
C_6_H_10_O	**29.0 ± 3.4**	179.5 ± 162.2	51.4 ± 12.8	61.6 ± 10.8
C_5_H_8_O_2_	**34.5 ± 2.9**	96.9 ± 24.1	34.9 ± 4.0	73.8 ± 14.1
C_6_H_8_O	56.5 ± 14.9	146.4 ± 20.5	**38.9 ± 9.0**	87.5 ± 16.1
C_6_H_12_O	**33.7 ± 18.1**	74.5 ± 38.7	34.5 ± 14.0	76.5 ± 13.3
C_5_H_10_O_2_	**24.2 ± 3.9**	97.8 ± 46.2	44.1 ± 7.7	86.6 ± 7.3
C_5_H_6_O_2_	**35.5 ± 10.0**	143.5 ± 38.8	40.9 ± 3.0	98.5 ± 16.6

**Table:** 

	Force RMSE (eV Å^−1^)			
	GO-MACE-23	TensorNet	NequIP	PaiNN
Aspirin	**0.21 ± 0.03**	0.43 ± 0.09	0.24 ± 0.04	0.50 ± 0.12
Ethanol	**0.32 ± 0.05**	0.69 ± 0.24	0.39 ± 0.02	0.66 ± 0.13
Malo.	**0.20 ± 0.02**	0.52 ± 0.11	0.24 ± 0.03	0.46 ± 0.04
Naphth.	**0.06 ± 0.01**	0.21 ± 0.07	0.10 ± 0.02	0.30 ± 0.05
Salicylic	**0.10 ± 0.01**	0.26 ± 0.08	0.12 ± 0.01	0.36 ± 0.08
Toluene	**0.17 ± 0.03**	0.39 ± 0.12	0.20 ± 0.03	0.46 ± 0.13
C_6_H_10_O	**0.62 ± 0.05**	1.14 ± 0.12	0.70 ± 0.08	1.28 ± 0.09
C_5_H_8_O_2_	**0.45 ± 0.04**	0.91 ± 0.16	0.52 ± 0.04	0.94 ± 0.05
C_6_H_8_O	**0.45 ± 0.05**	0.93 ± 0.18	0.56 ± 0.06	0.96 ± 0.10
C_6_H_12_O	**0.43 ± 0.06**	0.93 ± 0.19	0.53 ± 0.10	1.08 ± 0.07
C_5_H_10_O_2_	**0.41 ± 0.03**	0.87 ± 0.19	0.45 ± 0.02	0.91 ± 0.05
C_5_H_6_O_2_	**0.45 ± 0.02**	0.93 ± 0.18	0.47 ± 0.04	1.00 ± 0.08

These results highlight the importance of the MLIP architecture in capturing relevant atomistic information and transferring it beyond the training set. The extrapolation is not trivial and depends not only on the quality of the training data or the fit but also on the architecture itself. Notably, as shown in the SI, GO-MACE-23 has the lowest energy validation errors on the GO dataset, yet NequIP outperforms it for several rMD17 molecules in energy predictions. These results underscore the need for out-of-domain validation to fully assess model generalisation. Additionally, one could systematically investigate how the implementation choices of these GNNs, particularly in their atomic representations, influence their extrapolation capabilities, thereby enabling an *a priori* assessment of the performance of these MLIPs.^39,42,65^

### Transferability to chemical reactions

The long-term goal of molecular interatomic potentials is to describe entire reaction mechanisms, rather than just the reactants and products. MLIPs are increasingly being used to describe transition states of reactions in vacuum^66,67^ and in explicit solvent.^7^ While GO-MACE-23 will have “seen” various rearrangements, decarbonylation reactions, *etc.*, during iterative training,^20^ it has not been explicitly trained on molecular reaction mechanisms.

**Fig. 8:**
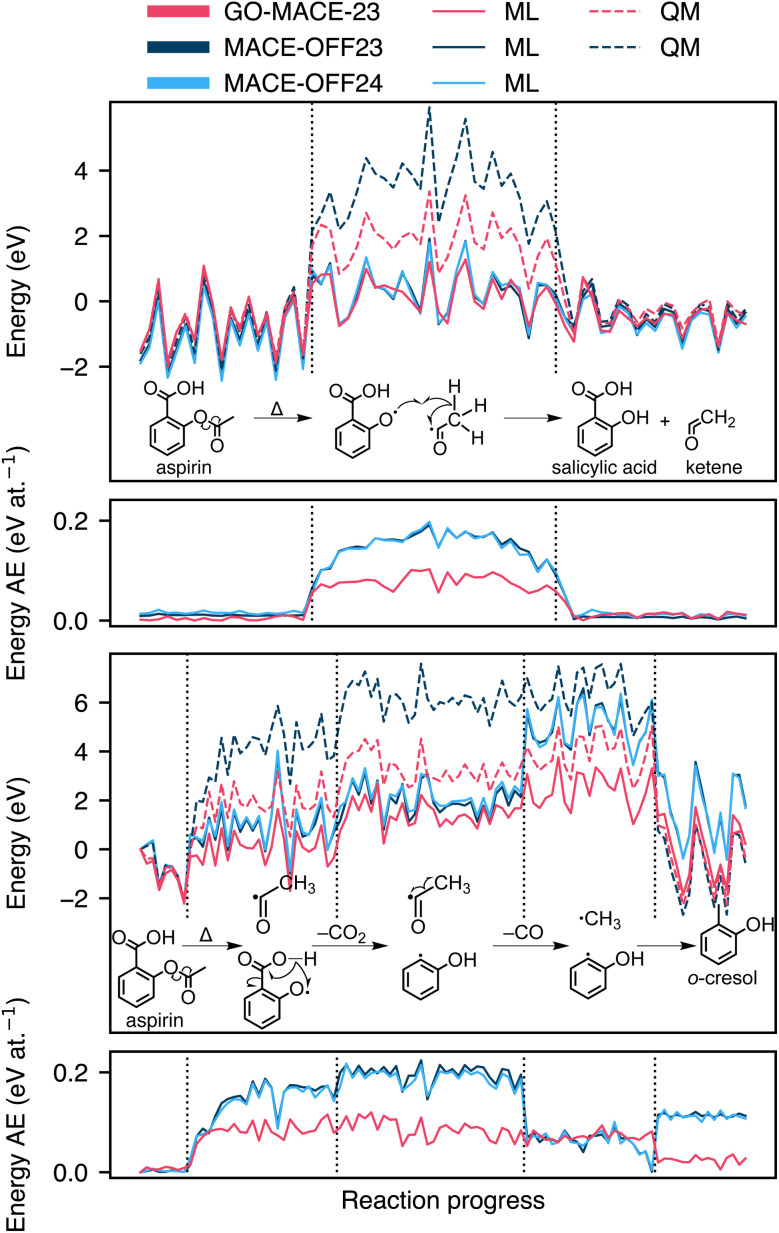
Energy profiles of two exemplary high-temperature molecular-dynamics simulations computed with GO-MACE-23, MACE-OFF23, MACE-OFF24, and their respective QM references. The MD trajectories are driven by GO-MACE-23 and maintained at 1500 K. The first panel describes a reaction pathway to produce salicylic acid and ketene (H_2_CCO) from aspirin. The third panel describes the decomposition of aspirin through a series of decarbonylations and decarboxylations to produce *o*-cresol. The second and fourth panels describe the difference between energies computed with ML and QM, for the first and second reactions, respectively, and expressed per atom.

We use GO-MACE-23 to run a series of MD trajectories of an aspirin molecule in a periodic simulation cell of 30 Å length, using the *NVT* ensemble at *T* = 1,500 K. We re-label snapshots from the trajectories using the DFT reference method of GO-MACE-23, as well as using both MACE-OFF24 variants and their DFT reference method. In Fig. 8, we report two reaction pathways for the thermally driven decomposition of aspirin in vacuum into radical species which then recombine forming different molecules.

The upper panels of Fig. 8 depict the formation of reactive ketene and salicylic acid, a process involving the breaking of an ester bond. The reverse reaction was first described in ref. 68. Both GO-MACE-23 and the MACE-OFF24 variants accurately capture the energetics of the reactants and products. However, they significantly underestimate the energy of the intermediates. Despite this underestimation, the predicted average energy of the intermediates remains higher than that of the more stable reactants or products. In addition, these MLIPs were not able to reproduce the energy of the isolated radicals. We note that proper treatment of radicals requires open-shell methods, *e.g.* coupled-cluster theory,^69,70^ or multireference approaches such as CASSCF,^71–74^ particularly for modelling processes like *cis*-to *trans*-isomerisations. Stocker *et al.*^75^ have previously discussed the limitations of MLIPs in accurately describing chemical reactions when radicals are not incorporated in the training data.

The lower panels of Fig. 8 illustrate the formation of an *o*-cresol molecule through a series of decarboxylation and decarbonylation steps. This reaction pathway shares the first set of radicals with the upper panel, with similar geometries, before developing into a different pathway. As with the previous pathway, all tested MLIPs underestimate the energy of the intermediate steps. The two models from the MACE-OFF24 family in particular overestimate the energy of the product system.

Furthermore, we test an earlier model version from the iterative training of GO-MACE-23: this version, denoted “iter-8” in ref. 20, was not trained on edge structures. We find that GO-MACE-23 outperforms its simpler counterpart, especially in describing radicals and the products (SI). This indicates that some of the edge structures—with different chemical functionalisation—included in later iterations have likely contributed some information relevant to gas-phase molecular reactions to the GO-MACE-23 training data.

We emphasise that the present case study is not aimed at fully assessing performance in reaction modelling—but rather as a challenging test that deliberately takes the MLIP models away from their training domains. These (very-) high-temperature MD trajectories are not guaranteed to find the overall most favourable pathway, and yet they end in chemically sensible molecules. Following these trajectories as explored by the MLIPs themselves, we probe the potential-energy landscape for a range of configurations different from those in the rMD17 and QM7-X sets. This test completes our series of progressively more challenging “zero-shot” evaluations of GO-MACE-23 outside of its domain of training.

## Conclusions

Located at the interface of materials and molecular modelling, graphene oxide offers an opportunity to connect these different domains of atomistic machine learning. In the present work, we have systematically assessed the zero-shot transferability of GO-MACE-23, an MLIP trained on data for GO, across relevant chemical benchmarks. We found good—perhaps surprisingly good—zero-shot performance compared to MACE-OFF24 a pre-trained model for molecular chemistry. The accuracy of both models decreases when describing reaction pathways, especially when radical species are involved.

Our study has tested the behaviour of recently proposed GNN MLIP models and their transferability, and we think that it can have implications for the future development of “foundational” models for atomistic simulations. Our results emphasise that including chemical reactivity in the training data is important in finding reaction pathways: in the process of building the GO-MACE-23 model,^20^ we have sampled this reactivity in high-temperature MD simulations, and a similar approaches have been taken, *e.g.*, for the bulk carbon–hydrogen^76^ and carbon–oxygen systems,^77^ as well as organic reactions in the condensed phase.^78^ We think that local-environment diversity will be as important as the chemical space coverage (*e.g.*, the number of chemical species) in defining future foundational models—this might include the addition of radical species (*cf.*Fig. 8) to the training data, either through very-high-temperature MD exploration or perhaps by explicitly involving “broken” bonds in the training protocol. Steps in this direction have been reported very recently.^79^

Despite its limitation to the three elements C, H, and O, the GO-MACE-23 model seems to provide a suitable starting point to study a wider range of chemistry-related questions than it was initially intended for, and we view this as a highly encouraging finding. We believe that together with improved data-generation strategies^22^ as well as suitable workflows and automation approaches,^80–83^ truly universal MLIPs for molecular systems, and for extended material structures built up from them, are coming within reach.

## Author contributions

C. B. M., Z. E.-M., and V. L. D. designed the research. K. A. G. carried out pilot studies, and C. B. M. and Z. E.-M. carried out the final numerical experiments. J. L. A. G. provided code and methodology for MLIP fitting. All authors contributed to discussions. C. B. M. and V. L. D. wrote the manuscript, and all authors reviewed and approved the final version.

## Conflicts of interest

There are no conflicts to declare.

## Supplementary Material

DD-004-D5DD00103J-s001

## Data Availability

Data supporting the present study, including analysis code snippets, are available at https://github.com/cbenmahm/GO-Zero-Shot. A copy has been archived in Zenodo and is available at https://doi.org/10.5281/zenodo.17183916. An archived version of GraphPES is available at https://zenodo.org/records/14956211. Supplementary information is available. See DOI: https://doi.org/10.1039/d5dd00103j.
