# Supplementary material for: Assessing zero-shot generalisation behaviour in graph-neural-network interatomic potentials
Source: Digit Discov. 2025 Sep 30;4(11):3389–99. doi: 10.1039/d5dd00103j (PMC12538557; doi:10.1039/d5dd00103j)
Supplement: DD-004-D5DD00103J-s001 [file DD-004-D5DD00103J-s001.pdf]

Supplementary Information (SI) for Digital Discovery.
This journal is © The Royal Society of Chemistry 2025

Supplementary Information for "Assessing zero-shot generalisation behaviour in graph-neural-network interatomic potentials"

Chiheb Ben Mahmoud,[1,*] Zakariya El-Machachi,[1] Krystian A. Gierczak,[1] John L. A. Gardner,[1] and Volker L. Deringer[1]

[1] *Inorganic Chemistry Laboratory, Department of Chemistry, University of Oxford, Oxford OX1 3QR, United Kingdom*

(Dated: September 23, 2025)

* chiheb.benmahmoud@chem.ox.ac.uk

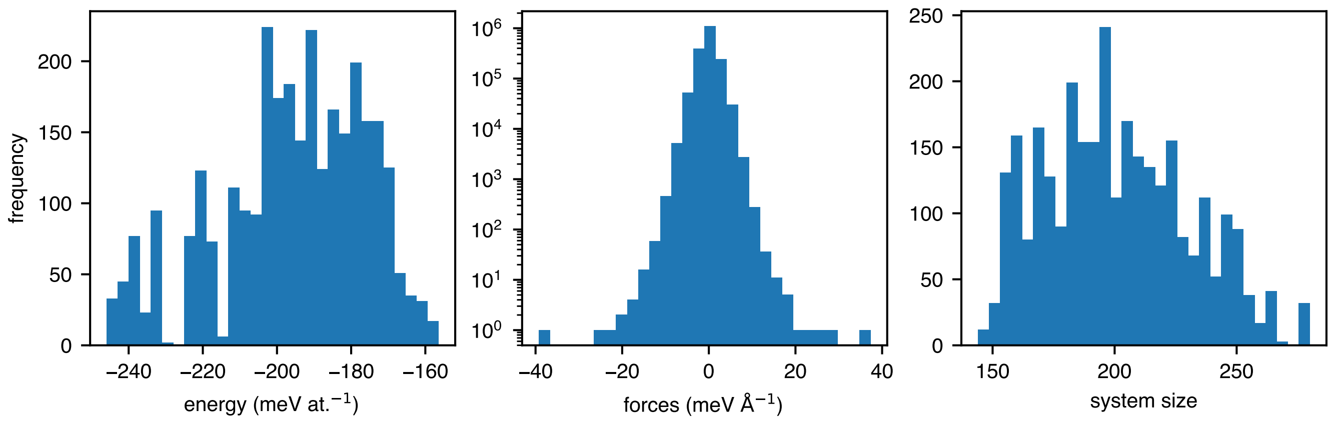

Figure S1: Distribution of the total energy (per atom), force components and system size of the GO training set.

I. TRAINING DETAILS FOR GO-MACE-23

An initial dataset was generated using the CASTEP+ML adaptive scheme [S1], which accelerates sampling relative to *ab initio* molecular dynamics. Twenty-five 10 ps trajectories at 300 K were performed across a 5×5 grid of O/C and OH/O ratios, yielding 820 seed structures. This dataset was expanded through iterative rounds of MACE-driven MD at increasing temperatures (600–1500 K), where candidate structures were filtered to remove nonphysical configurations (interatomic distances <0.5 Å; coordination numbers >6 or force components >50 eV Å^{-1}) and farthest-point sampling on smooth overlap of atomic positions (SOAP) descriptors [S2] was applied to ensure diversity. In total, 2,200 additional structures were added, resulting in a final training set of 3,013 configurations (≈605,000 atoms) and a test set of 800 configurations (≈163,000 atoms), including both condensed-phase systems and gaseous fragments (OH, H_2O, CO, CO_2, etc). In Fig. S1, we show the distribution of the total energy, force components, and system sizes in the GO training set. The latter contains 1.78% hydrogen, 71.66% carbon, and 26.55% oxygen atoms.

MACE models [S3] were trained with a Huber loss, a batch size of 30, maximum 2000 epochs with an early stopping criterion, energy-to-force weighting in the loss function of 1000:100, and a learning rate of 0.001.

Full details of the dataset curation and fitting protocol can be found in Ref. [S4].

II. HYPERPARAMETERS OF GRAPH-NEURAL NETWORKS TRAINED ON THE GRAPHENE OXIDE DATASET

In Table S1, we report the main hyperparameters of the GNNs trained on the GO dataset. For PaiNN, NequIP and TensorNet (see references in the main text), we used a radial cut-off of 3.7 Å, and targeted ≈ 150,000 parameters. These choices were made to ensure comparable model sizes while maintaining diversity in architectural design across different GNNs. We also include the main hyperparameters of `GO-MACE-23` and the `MACE-OFF` models used herein.

Table S1: Hyperparameters of GNNs used in this work. Channels per layer uses the `e3nn` [S5] notations to refer to the number, order and parity of spherical harmonics.

MLIP	cutoff (Å)	Layers	Channels per Layer	Radial Features
`GO-MACE-23`	3.7	2	128x0e + 128x1o	8
`MACE-OFF23`(L)	5.0	2	192x0e + 192x1o + 192x2e	8
`MACE-OFF24`(M)	6.0	2	128x0e + 128x1o	8
PaiNN	3.7	3	64	16
NequIP	3.7	3	32x0e + 32x0o + 32x1e + 32x1o + 32x2e + 32x2o	16
TensorNet	3.7	2	64	32

III. TRAINING PROTOCOL FOR GRAPH-BASED MLIPS

We use GraphPES to train four graph-based MLIPs: TensorNet, NequIP, and PaiNN, on the GO dataset. All models are trained with the same protocol. The optimisation is guided by a combined loss comprising a per-atom energy RMSE and a force RMSE, with equal weights. The batch size is 64. The training is performed for a maximum of 10,000 epochs, alongside early stopping with a patience of 150 epochs to prevent overfitting.

IV. VALIDATION ERROR OF GRAPH-NEURAL NETWORKS TRAINED ON THE GRAPHENE OXIDE DATASET

In Table S2, we report the energy and force validation RMSE of MLIP models trained on the graphene dataset.

Table S2: Validation errors on energy and forces for the different MLIPs trained on the graphene oxide dataset

MLIP	Energy RMSE (meV at.$^{-1}$)	Forces RMSE (eV Å^{-1})
GO-MACE-23	1.8	110
GO-MACE-23 L=0	2.5	157
GO-MACE-23 L=2	2.3	125
PaiNN	3.0	207
TensorNet	2.3	182
NequIP	2.7	182

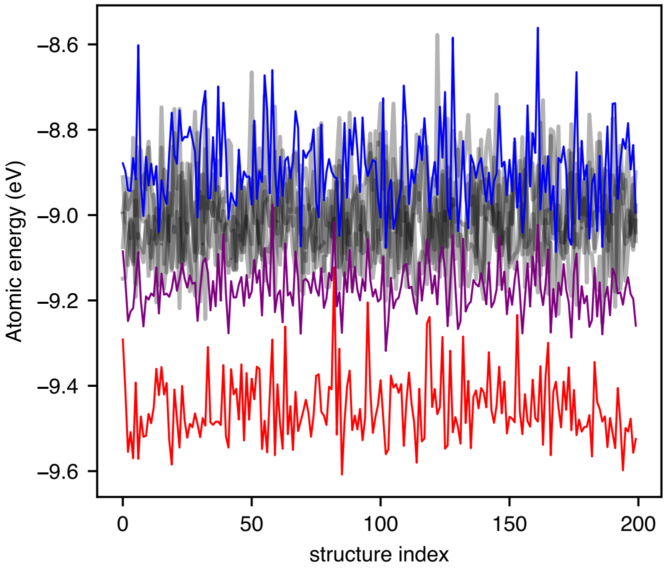

Figure S2: Atomic energies as predicted by `GO-MACE-23` for 200 structures. Red, blue and purple represent the atomic energy of the carbon in the aromatic ring directly connected to the methyl group, the methyl carbon, and their average, respectively. The black curves represent the atomic energies of the remainder of the carbon atoms in the aromatic ring.

V. ATOMIC ENERGIES OF TOLUENE

In Fig. S2, we show the local atomic energies of 200 snapshots from the toluene trajectory, as predicted by `GO-MACE-23`.

VI. ROOT MEAN SQUARED DISPLACEMENT OF MOLECULES RELAXED WITH MLIPS AND DFT

In Table S3, we report the root-mean-square displacement of rMD17 and QM7-X molecules relaxed with `GO-MACE-23` and its reference DFT level of theory.

Table S3: Root mean squared displacement of molecules

molecule	RMSD (Å)
naphthalene	0.23
toluene	0.17
malonaldehyde	0.25
$C_5H_8O_2$	0.22
$C_6H_{12}O$	0.28
$C_6H_{10}O$	0.17

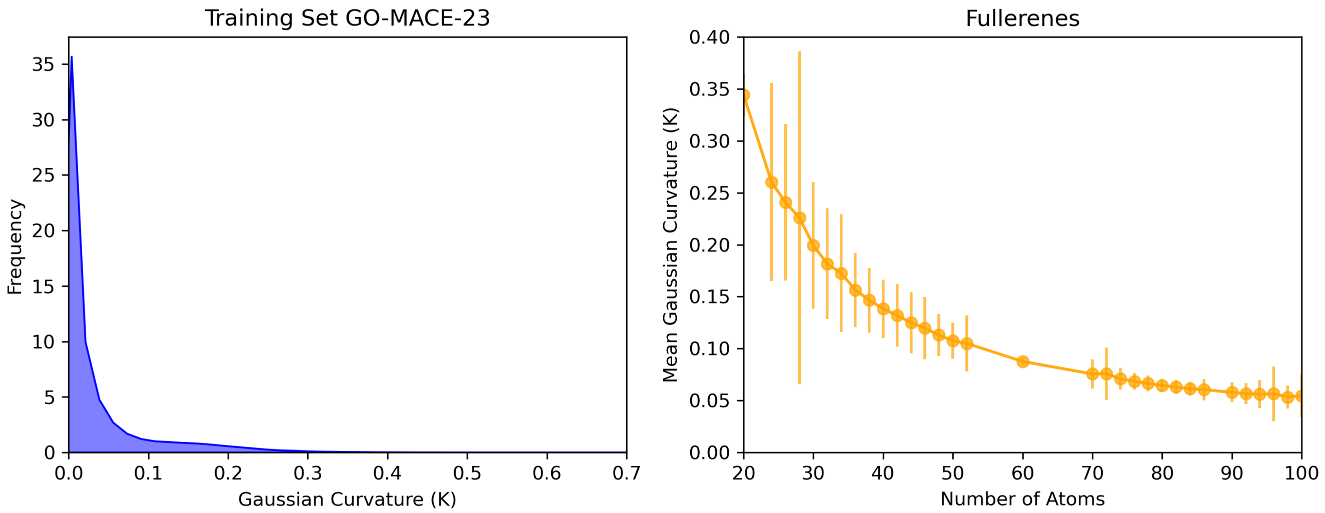

Figure S3: Kernel density estimate of local Gaussian curvature of `GO-MACE-23` training dataset (left). Also plotted is the mean local Gaussian curvature for the fullerene dataset (right) with standard deviations.

VII. CURVATURE ANALYSIS

In order to understand why `GO-MACE-23` is relatively accurate at predicting fullerene properties, we posit that the underlying curvature within the `GO-MACE-23` training set overlaps with the curvature displayed in fullerenes. We approach this by computing the local Gaussian curvature for carbon atoms which comprise the "graphene" backbone in all structures in the training set. Gaseous species and O atoms were explicitly removed as a result for the purposes of this analysis.

The Gaussian curvature, K, was evaluated for each atom by fitting a local quadratic surface to its neighbourhood, following the procedure outlined in Algorithm 1. Neighbourhoods were identified using the `ASE neighbor_list` function with a cutoff distance of 1.85 Å, which includes first nearest-neighbours connected via typical C–C bonds in sp^2 networks. Atoms with less than 3 neighbours were not accounted for in the curvature calculations. For each central atom, the positions of its neighbours were first translated so that the central atom lay at the origin. A principal component analysis (PCA) was then applied to define a local orthonormal coordinate system, with the u–v plane representing the best-fit tangent plane and w the out-of-plane coordinate.

The $w(u, v)$ surface was approximated as a second-order polynomial:

$$w(u, v) = au^2 + b\,uv + cv^2 + d\,u + e\,v + f_0$$

From the fitted coefficients, the first and second derivatives of w at $(u, v) = (0, 0)$ were computed to obtain the coefficients of the first and second fundamental forms:

$$E = 1 + f_u^2, \quad F = f_u f_v, \quad G = 1 + f_v^2$$

$$L = f_{uu}, \quad M = f_{uv}, \quad N = f_{vv}$$

The local Gaussian curvature was then calculated as:

$$K = \frac{LN - M^2}{EG - F^2}$$

which measures the product of the principal curvatures and is independent of the surface's embedding in three-dimensional space.

Algorithm 1 Computation of Gaussian curvature from atomic coordinates

Require: Atomic structure $\mathcal{A}$ with atom positions $\mathbf{r}_i$
Select atoms of interest (e.g., carbon atoms in fullerene)
Compute centroid $\mathbf{c}$ of selected atoms
Perform PCA on positions to obtain local orthonormal basis $(\mathbf{u}, \mathbf{v}, \mathbf{w})$
for each atom i **do**
  Identify neighbouring atoms $\mathcal{N}(i)$ via cutoff or bonding criterion
  Fit a local quadratic surface $z = ax^2 + bxy + cy^2 + dx + ey + f$ to the neighbourhood in local coordinates
  Compute the first and second fundamental forms from fitted coefficients
  Evaluate Gaussian curvature K_i at the atom using

$$K_i = \frac{LN - M^2}{EG - F^2}$$

  where (E, F, G) and (L, N, M) are first and second fundamental form components
end for
return $\{K_i\}$, the per-atom Gaussian curvature values

Interpretation of K is as follows:

- $K > 0$: the surface is locally convex or concave in all directions, e.g., spherical cap regions such as in fullerene cages or pentagons in defective graphene
- $K = 0$: the surface is locally flat or cylindrical, as in ideal graphene sheets or nanotubes (hexagons).
- $K < 0$: the surface is saddle-shaped, with curvature of opposite sign in orthogonal directions, typically found with heptagon rings in defective graphene.

In this work, the distribution of K values for the `GO-MACE-23` dataset shows a significant fraction of positively curved sites, overlapping with the large fullerene ($>$ 40 atoms) distribution (Fig. S3). This suggests that the training set already contains many atomic environments with curvature characteristics similar to those in larger fullerenes, providing a geometric basis for the observed accuracy of `GO-MACE-23` on fullerene property predictions due to the locality of `GO-MACE-23` having a perceptive field of 3.7 Å$\times$ 2 layers.

We must note that the omission of dangling bonds, sp and sp^3 carbon atoms, and O atoms means that whilst the curvatures reported are not indicative of the *actual* curvature, we emphasise the overlap in local motifs which are important in the geometric overlap of both fullerenes and GO.

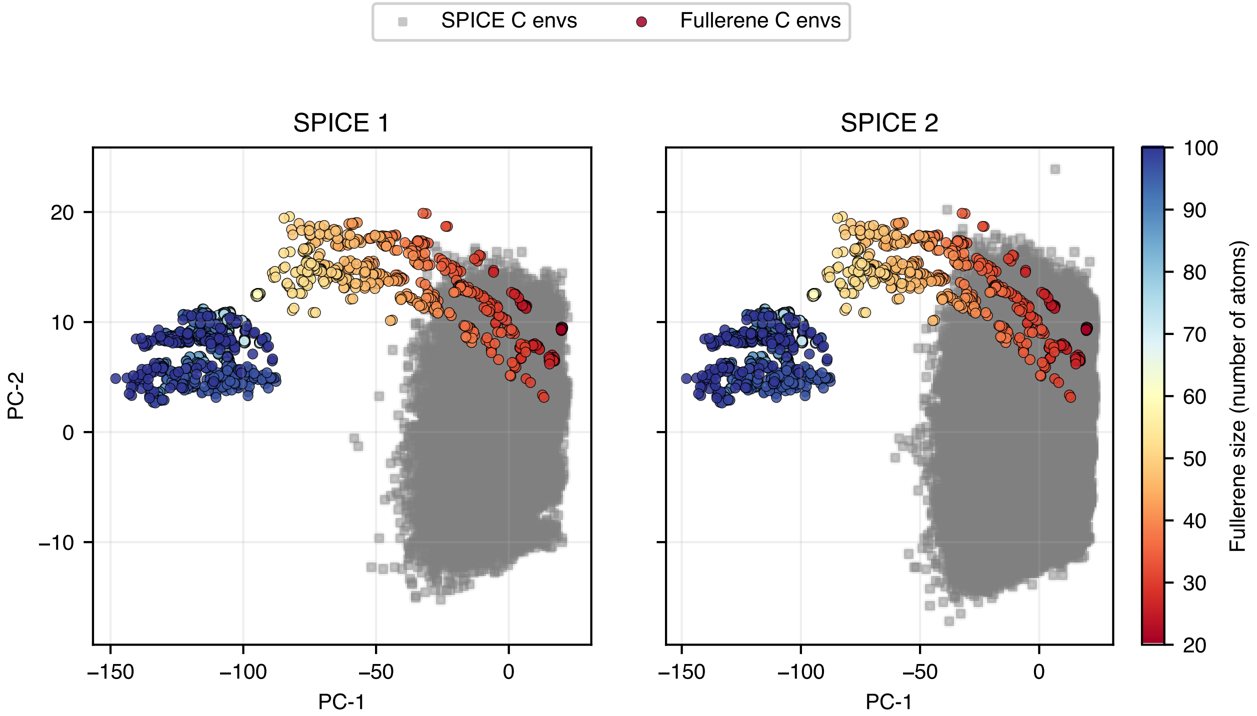

Figure S4: Visualising the structural space of the SPICE 1 and 2 datasets, and the fullerenes of sizes ranging between 20 and 100 atoms. We show a two-dimensional embedding of the atomic SOAP descriptors. The overlap between the SPICE datasets and the fullerenes only concerns small fullerenes (number of atoms < 40 for SPICE 1 and < 50 for SPICE 2).

VIII. PERFORMANCE ON FULLERENES

In Fig. S4, we present PCA mapping of SOAP descriptors computed for the fullerene dataset alongside subsets of the SPICE 1 and 2 datasets. For the SPICE datasets, we restrict ourselves to configurations containing more than 20 carbon atoms. The SOAP descriptors for carbon environments have been computed using the `dscribe` [S6] implementation with $r_{\mathrm{cut}} = 6.0$, $n_{\mathrm{max}} = 8$, and $l_{\mathrm{max}} = 6$. It can be seen from the figure above that while the carbon environments of the largest fullerenes (> 75 carbon atoms) do not seem to overlap with either SPICE 1 or 2 at all, the extension of SPICE 2 relative to SPICE 1 results in slightly greater overlap with fullerenes of sizes in the range 40–50 atoms, thereby providing a plausible explanation of the good performance of `MACE-OFF24` on these structures.

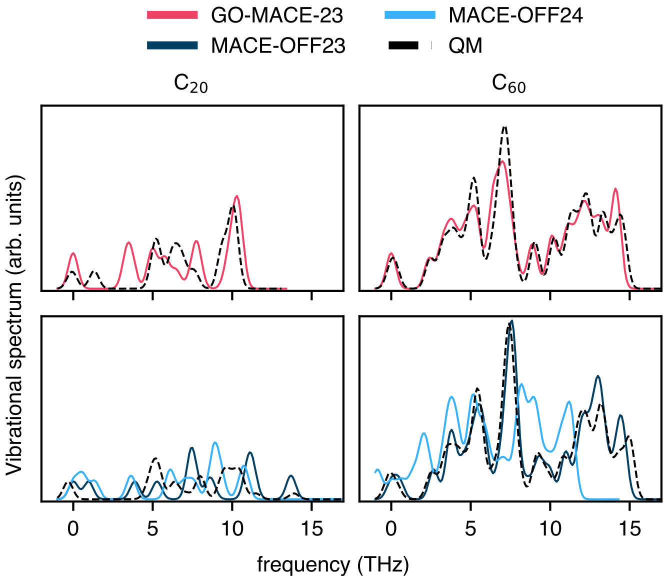

Figure S5: Vibrational spectra of C_{20} and C_{60} from `GO-MACE-23`, `MACE-OFF`s, and their corresponding DFT levels of theory.

IX. VIBRATIONAL SPECTRA OF FULLERENES

In Fig. S5, we show the vibrational spectra of C_{20} and C_{60} computed with `GO-MACE-23`, `MACE-OFF`s, and their corresponding DFT references. `GO-MACE-23` yields reasonable accuracy for both fullerenes. `MACE-OFF24` reproduces well the spectrum of C_{60}. Both `MACE-OFF` models do not correctly predict the spectrum of C_{20}.

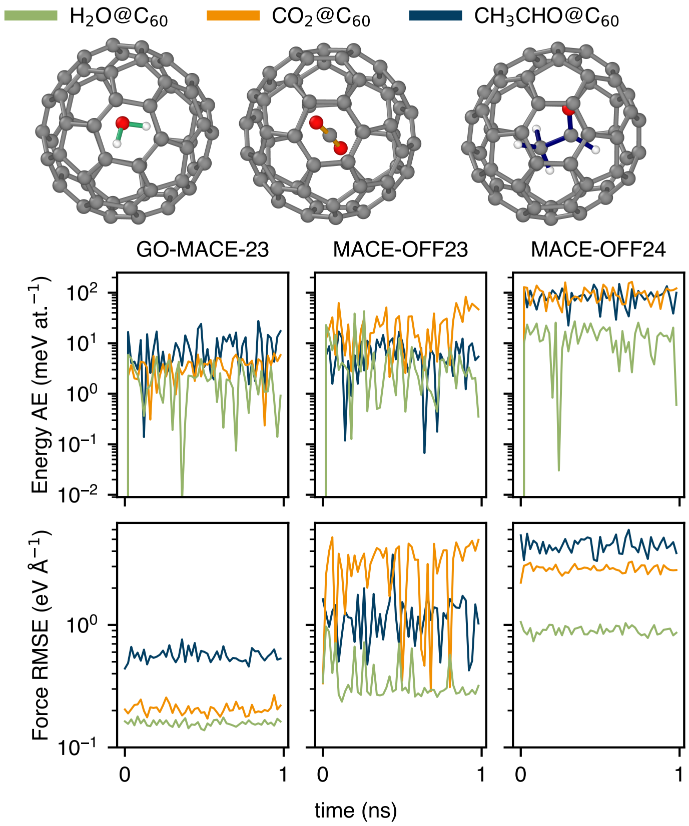

Figure S6: Evolution of energy and force RMSE, between `GO-MACE-23` predictions and the corresponding DFT level of theory (left column) and `MACE-OFF`s and their corresponding DFT level of theory (centre and right columns), per snapshot of 1 ns trajectories at 500 K of H_2O, CO_2, and CH_3CHO encapsulated in a C_{60} fullerene. The trajectories are driven by `GO-MACE-23`.

X. ENCAPSULATED MOLECULES IN C_{60} CAGES

In Fig. S6, we show the evolution of energy and force errors of three encapsulated reactions: $H_2O@C_{60}$, $CO_2@C_{60}$, and $CH_3CHO@C_{60}$, as predicted by `GO-MACE-23` and `MACE-OFF`. The first species has already been discussed in the main text and reported here for comparison. The latter two have not been reported, to the best of out knowledge, in literature and are just a thought experiment for now. Both MLIPs produce energies compatible with DFT, however, `MACE-OFF` is not able to recover the correct forces for these trajectories, with force RMSE exceeding 1 eV $Å^{-1}$ in the $CO_2@C_{60}$ hypothetical species.

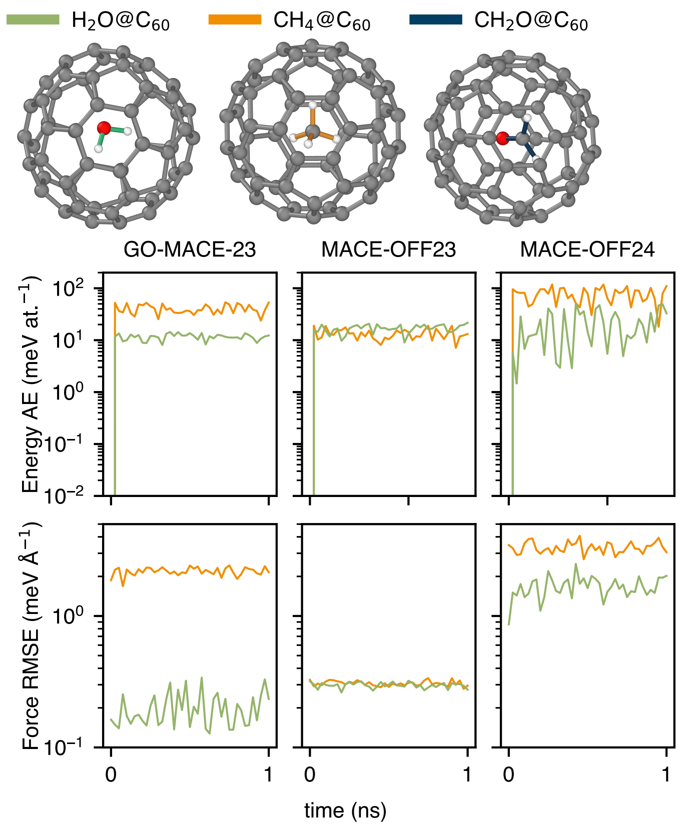

Figure S7: Evolution of energy and force RMSE, between GO-MACE-23 predictions and the corresponding DFT level of theory (left column) and MACE-OFFs and their corresponding DFT level of theory (centre and right columns), per snapshot of 1 ns trajectories at 500 K of H_2O, CH_4, and CH_2O encapsulated in a C_{60} fullerene. The $CH_2O@C_{60}$ trajectory failed after the first step. The trajectories are driven by MACE-OFF23.

XI. ENCAPSULATED MOLECULES IN C_{60} CAGES DRIVEN BY MACE-OFF23

In Figs. S7 and S8, we show the evolution of energy and force errors of the six encapsulated reactions as predicted by GO-MACE-23 and MACE-OFF. The trajectories are driven by MACE-OFF23. The $CH_2O@C_{60}$ failed after the first timestep. We notice that GO-MACE-23 is more accurate in recreating the energies and forces for $H_2O@C_{60}$ and $CO_2@C_{60}$, while MACE-OFF23outperforms in $CH_3CHO@C_{60}$ and $CH_4@C_{60}$.

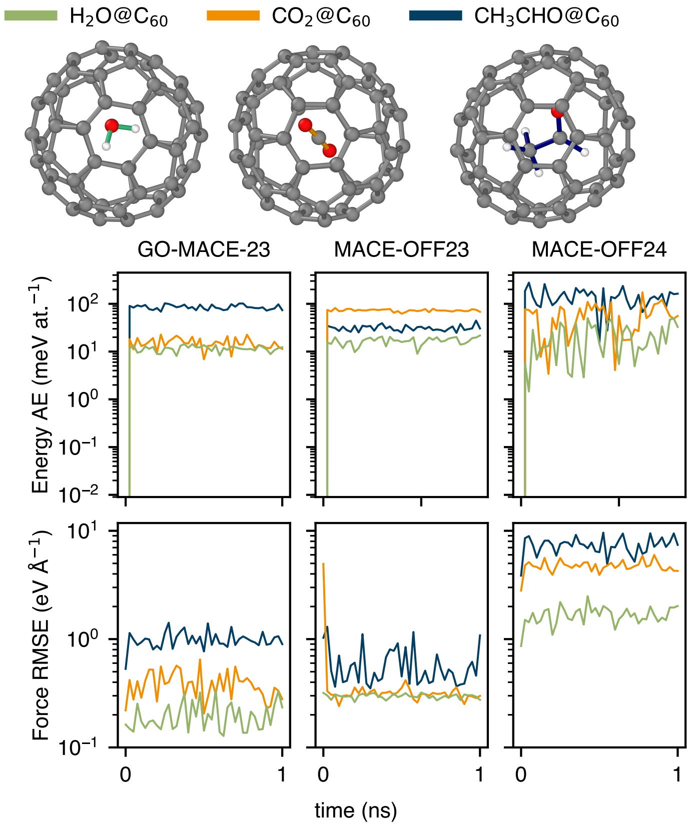

Figure S8: Evolution of energy and force RMSE, between `GO-MACE-23` predictions and the corresponding DFT level of theory (left column) and `MACE-OFF`s and their corresponding DFT level of theory (centre and right columns), per snapshot of 1 ns trajectories at 500 K of H_2O, CO_2, and CH_3CHO encapsulated in a C_{60} fullerene. The trajectories are driven by `MACE-OFF23`.

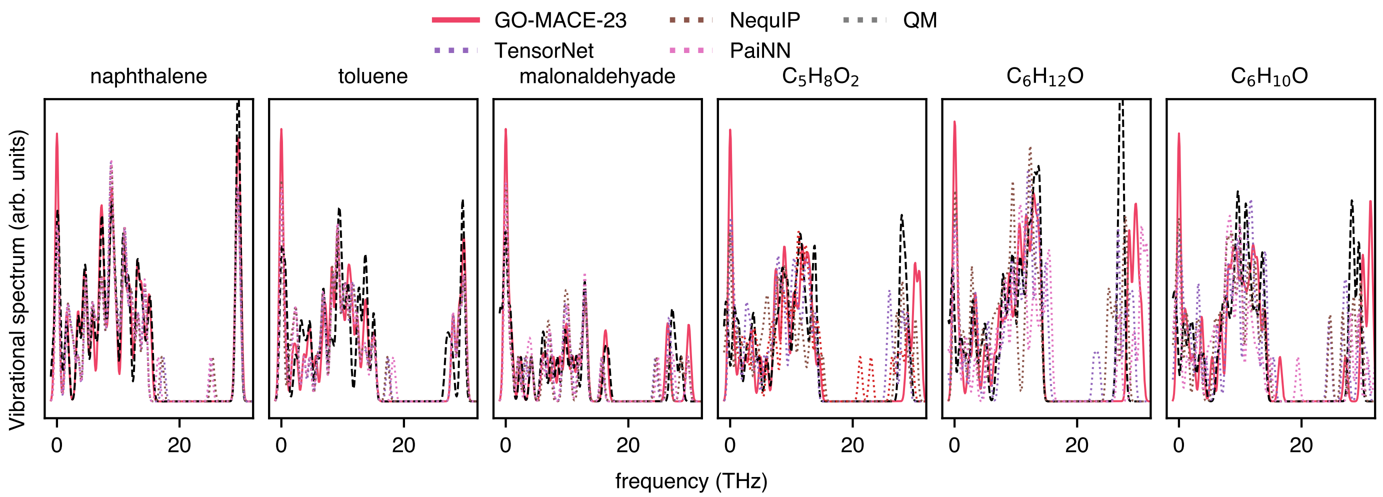

Figure S9: Vibrational spectra obtained from GNNs trained on the GO dataset and their DFT reference.

XII. VIBRATIONAL SPECTRA OF RMD17 AND QM7-X FROM GNNS

In Fig. S9, we report the vibrational spectra obtained from the GNNs trained on the GO dataset and discussed in the main text: NequIP, PaiNN, and TensorNet. We also compare to spectra from `GO-MACE-23` and its DFT reference. These MLIPs yield reasonable accuracy in the low frequency regime. However, they are not able to replicate the high frequency spectrum.

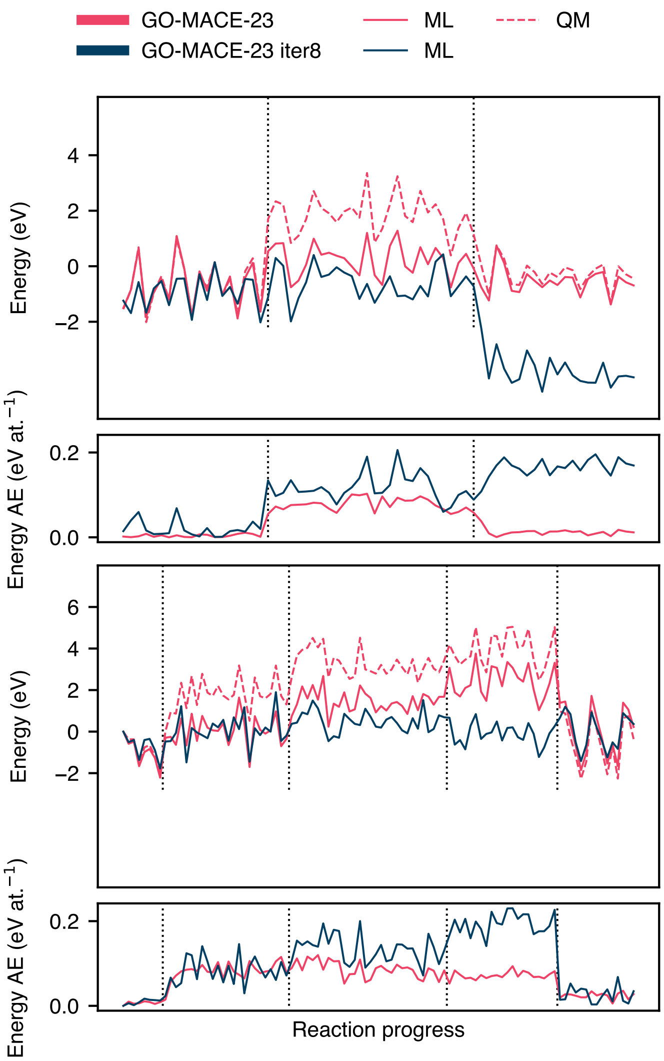

Figure S10: Energy profiles of two exemplary high-temperature molecular-dynamics simulations computed with `GO-MACE-23` and `GO-MACE-23iter8`, an early version of `GO-MACE-23` that was not trained on edges.

XIII. REACTIONS WITH AN INTERMEDIATE MODEL

In Fig. S10, we show the energy profiles of two reaction pathways, driven by `GO-MACE-23`, shown in the "Transferability to chemical reactions" section of the main text. Then, the trajectories are recomputed with `GO-MACE-23`, an early version of `GO-MACE-23` that was not trained on edges (`GO-MACE-23 iter8`), and their corresponding DFT level of theory. We find that `GO-MACE-23iter8` yields higher prediction errors compared to `GO-MACE-23`, which hints towards the importance of including reactive edges in the training MLIPs for chemistry applications.

[S1] T. K. Stenczel, Z. El-Machachi, G. Liepuoniute, J. D. Morrow, A. P. Bartók, M. I. J. Probert, G. Csányi, and V. L. Deringer, The Journal of Chemical Physics **159**, 044803 (2023).
[S2] A. P. Bartók, R. Kondor, and G. Csányi, Physical Review B **87**, 184115 (2013), publisher: American Physical Society.
[S3] I. Batatia, D. P. Kovacs, G. Simm, C. Ortner, and G. Csanyi, in *Advances in neural information processing systems*, Vol. 35, edited by S. Koyejo, S. Mohamed, A. Agarwal, D. Belgrave, K. Cho, and A. Oh (Curran Associates, Inc., 2022) pp. 11423–11436.
[S4] Z. El-Machachi, D. Frantzov, A. Nijamudheen, T. Zarrouk, M. A. Caro, and V. L. Deringer, Angewandte Chemie International Edition , e202410088 (2024).
[S5] M. Geiger and T. Smidt, "e3nn: Euclidean Neural Networks," (2022), version Number: 1.
[S6] J. Laakso, L. Himanen, H. Homm, E. V. Morooka, M. O. J. Jäger, M. Todorović, and P. Rinke, The Journal of Chemical Physics **158**, 234802 (2023).
